# Recognizability Affects the Processing of Facial Gender Information

**DOI:** 10.1002/brb3.71826

**Published:** 2026-10-06

**Authors:** Szabolcs Sáringer, András Benyhe, Péter Kaposvári

**Affiliations:** ^1^ Department of Physiology, Albert Szent‐Györgyi Medical School University of Szeged Szeged Hungary

**Keywords:** EEG, face processing, facial gender, MVPA

## Abstract

**Purpose:**

The different aspects of a face, like sex, gender, or identity, can be decoded from the cortical patterns related to its processing. Many studies have investigated this phenomenon with similar outcomes. These studies usually utilize a low number of facial identities and high repetition numbers, potentially affecting the recognizability of a face, thus altering the processing. We propose that this commonly employed paradigm influences cortical patterns associated with features seemingly unrelated to identity.

**Method:**

In the first experiment, we adapted common paradigm parameters from previous studies using a few identities and a high presentation number for decoding facial gender. In the second experiment, the identity‐presentation ratio was switched.

**Findings:**

This change resulted in a narrower time window where facial gender related cortical patterns were detected. Decoding accuracy was also diminished, yielding lower values and suggesting a reduced signal‐to‐noise ratio in the cortex. After expanding the sample size to balance male and female participants, we identified shared cortical patterns related to facial gender processing both within the population and across gender‐based subpopulations.

**Conclusion:**

These results provide further evidence that exposure time to a singular face affects its processing and suggest that previous findings on perceived gender information decoding were likely influenced by the experimental paradigms employed.

## Graphical abstract

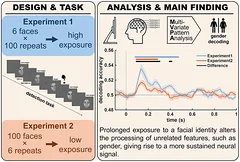

## 1 Introduction

Fast and accurate face recognition is a crucial part of social life. It has been established that this process is tied to the hierarchical system of the ventral visual pathway (Axelrod and Yovel 2012; Duchaine and Yovel 2015; Zhen et al. 2013). Both behavioral (Barragan‐Jason et al. 2013) and neural evidence (Goffaux and Dakin 2010) suggest that different aspects of facial information processing are distributed across the ventral stream both spatially and temporally.

One such aspect is the perceived sex or gender of the observed face. Note that the literature is often ambiguous regarding the studied variable, that is, sex versus gender. Throughout the text, we use “sex” to describe anatomical and physical facial features, while we use “gender” when these features are not directly measured, and participants and stimulus models have provided gender identities.

Participants can discriminate between facial sexes with high accuracy (Wild et al. 2000). Furthermore, behavioral data suggest a female advantage in face processing (Cellerino et al. 2004; Østergaard Knudsen et al. 2021), a possible hint that the mechanism might differ across genders (Proverbio et al. 2006). Besides the behavioral evidence, it was observed that face‐gender information activates the core and extended face network (Kaul et al. 2011; Podrebarac et al. 2013) and follows its hierarchical structure. For example, previous studies show that facial gender information is processed prior to identity (Besson et al. 2017; Dobs et al. 2019), although some suggest the processes might run in parallel (Rossion 2002).

It has also been established that the processing of familiar and unfamiliar faces differs in their neural signatures (Ambrus et al. 2019; Dobs et al. 2019; Kovács 2020). Particularly, the degree of familiarity, or the experience leading to familiarity, can determine the cortical representation of a given face. The level of familiarity is greatly affected by the experienced visual variance in the face and the semantic information associated with said face. Based on this, previous studies implemented the familiarization process on different levels: mere visual familiarization provides small variance and no semantic information. In contrast, personal familiarization can result in greater effects (Ambrus et al. 2021). Recent papers also suggest that faces activate the same networks to different extents depending on the level of familiarity (Kovács 2020).

Many studies have observed the underlying structure of the face‐processing network extending from the primary visual cortex to the fusiform face area. A growing number of studies investigate the temporal dynamics of the process using M/EEG‐based multivariate pattern analysis (MVPA) or representational similarity analysis (RSA) techniques (Ambrus et al. 2019, Ambrus et al. 2021; Dobs et al. 2019; Nemrodov et al. 2018). MVPA and RSA analyses allow us to observe the temporal dynamics of several features simultaneously. Furthermore, these techniques also allow identifying shared cortical patterns across participants or experiments (Ambrus 2024; Kaplan et al. 2015).

The results among the articles are consistent regarding early decoding differences, suggesting a short latency in the process. Different facial information (e.g., sex, identity, age) is coded in the cortical EEG pattern and can be distinguished as early as 50 ms, but generally speaking, most studies find a latency no longer than 100 ms. This is also consistent with behavioral results (short reaction times) and other imaging techniques (ECoG) where face‐related neural signals are detectable; for example, the activation of face‐selective areas in the inferotemporal cortex can be detected as early as 130 ms (Schrouff et al. 2020). A major disparity between the studies is the time window related to specific facial features and how long the aspect‐specific cortical patterns are maintained. Several of the above‐mentioned studies found 500–800 ms long windows. A common feature among these studies is that they used a low number of facial identities with high repetition numbers and usually investigated several facial features simultaneously, like sex or gender, identity, and familiarity. Although there is a great difference in the degree of familiarity, we can argue that mere exposure to unfamiliar faces will result in a familiarity effect (Ambrus et al. 2021). This experimental setup can familiarize the seemingly “unfamiliar” facial stimuli during the experiment. Although the difference in the level of familiarity is undeniable in these studies, unfamiliar faces become recognizable with high repetition numbers.

A study, similar to the ones already mentioned, investigated the emergence of identity‐specific cortical patterns over time. In contrast with many other publications, their goal only involved the observation of one facial feature (identity), and their methods included a high number of identities (91) with a relatively low number of repetitions (4) of one identity (Vida et al. 2017). Their findings were limited to a short time window (100–400 ms) despite the strong similarities with other RSA/MVPA studies. This raises the question of whether subjects are familiarized with faces during the stimulus exposure and how this low level of familiarity due to repeated exposure affects the temporal dynamics of face processing.

Here, we argue that the processing of faces changes over time due to repeated exposure, and this phenomenon affects seemingly unrelated facial features such as facial gender processing. To investigate the effect of visual exposure, we conducted two experiments. In Experiment 1, we set our goal to observe the findings of previous MVPA/RSA studies by adapting a paradigm with a high number of repeated displays (100×) of six identities and decoding face‐gender information from the related EEG activity. In Experiment 2, we switched the parameters by using 100 identities and a low number of repetitions (6×) for individual faces. The results of Experiment 2 indicate that reducing the recognizability of faces in a paradigm changes the temporal aspects of the cortical signal of facial gender processing.

Since the literature lacks similar paradigms, Experiment 2 was extended to further elaborate on gender processing. The balanced number of participants in the female and male subpopulations allows us to find similar or shared cortical patterns within the whole population, within a subpopulation, and between male and female subpopulations.

## 2 Materials and Methods

### 2.1 Experiment 1

#### 2.1.1 Participants

In Experiment 1, 25 healthy volunteers (17 females and 8 males [self‐reported], mean age ± SD of 23.85 ± 1.26 years) with normal or corrected‐to‐normal vision participated. The sample size was determined based on previous publications employing similar methodologies. All participants gave written informed consent before registration, and the study protocol was approved by the Hungarian National Center for Public Health and Pharmacy (NNGYK/49248‐3/2024).

#### 2.1.2 Stimuli

One hundred stimuli were selected from the Chicago Face Database (CFD; Ma et al. 2015), balanced between genders (50 males, 50 females, all identifying as White), with neutral facial expressions. The images were centered, cropped into an oval shape, and presented in grayscale on a gray background. In Experiment 1, six faces (3 females and 3 males) were selected randomly for each participant.

The variance between the stimuli was evaluated using the structural similarity index measure (Wang et al. 2004). SSIM ranged between 0.89 and 0.94 between images, with an average of 0.91 (Figure 1).

**FIGURE 1:**
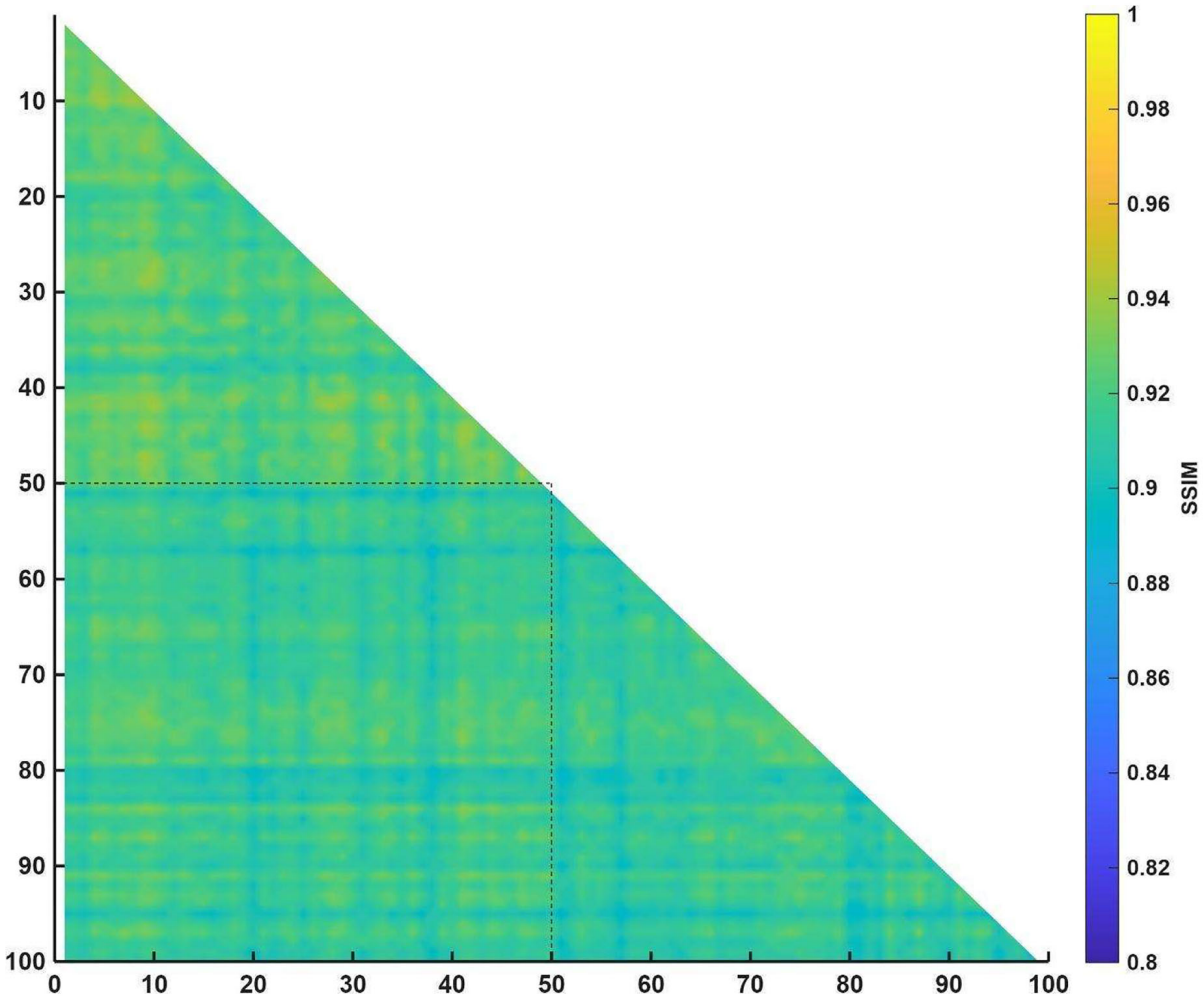
Structural similarity index measure between stimuli. The first 50 images were of female faces, while the last 50 were of male faces. The average SSIM between pictures was above 0.9.

*A note on gender labels*: In the CFD, gender labels are self‐reported by the models, and for our analyses we used the same labels for training and cross‐validation of the machine‐learning algorithms. Although a lot of variance in facial geometry is dependent on biological sex, and could be argued to drive perception, we could not use sex as a label, since this information was not explicitly available. Furthermore, evidence suggests that perceived gender correlates with facial feature components orthogonal to sexual dimorphism (Komori et al. 2011). Based on the CFD norming data (see Ma et al. 2015; http://www.chicagofaces.org/), the raters’ perception of facial gender matched the original labels above 95% for all of our stimuli. Since the two constructs largely overlap in the database, it is difficult to tell sex‐ and gender‐dependent effects apart in the EEG data. Therefore, to avoid any confusion, we will refer to the stimulus and perception qualities as gender and perceived gender, based on the original labels.

#### 2.1.3 Procedure

Participants were seated approximately 50 cm from the screen and instructed that they were going to see a sequence of faces. The stimuli were presented using Psychtoolbox, MATLAB 2023 (Kleiner et al. 2007). They were also tasked with indicating the appearance of a target stimulus in the sequence with a button press. In target trials, the face stimulus was presented in a tilted manner (15° anticlockwise rotation; note that the task does not require conscious attention to the gender or identity of the stimuli). The purpose of the target stimuli was to maintain the participants' attention throughout the image sequence. The EEG data of the target stimuli were excluded and not analyzed later.

All participants were presented with 600 stimuli (six faces and 100 repetitions), of which 10% were target stimuli, thus resulting in 540 non‐target stimuli for the subsequent analysis. A fixation cross was displayed at the center of the screen, which remained visible throughout the whole stimulus presentation. Stimuli (non‐target and target) were presented for 500 ms, followed by a jittered intertrial interval of 800–1200 ms (Figure 2). The whole sequence lasted approximately 15 min.

**FIGURE 2:**
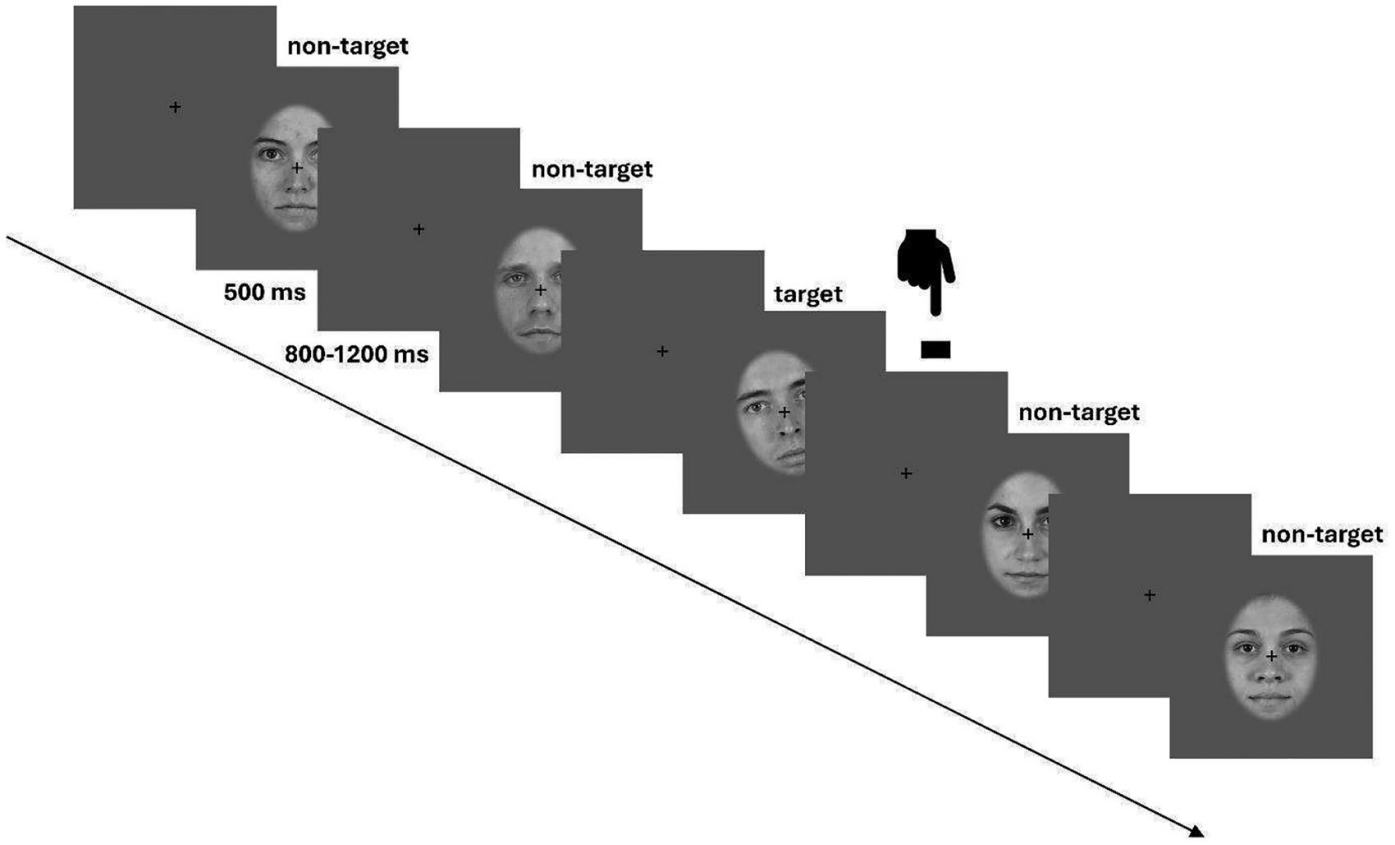
Stimulus presentation and structure.

#### 2.1.4 EEG Acquisition and Preprocessing

EEG data were recorded using a 64‐channel Biosemi Active II System with a sampling rate of 512 Hz. Alongside the EEG, a four‐channel EOG was recorded from the outer canthi of the eyes and above and below the left eye.

EEG preprocessing was implemented in MATLAB (2024a) using custom‐written scripts and the FieldTrip toolbox (Oostenveld et al. 2010). First, a bandpass filter was applied to the raw data between 2 and 40 Hz. Then, eye‐movement and blink artifacts were removed using a denoising source separation algorithm (Särelä and Valpola 2005). We trained the algorithm on blink events using pseudotrials centered on EOG peaks. These components were then removed from the EEG data. Epochs were defined in the continuous data from 0.5 s before stimulus presentation to 1.2 s after. The data were resampled to 100 Hz (resulting in 10 ms temporal resolution) and baselined to the prestimulus window of −0.3 to −0.1 s. Trials with unusually high variance were removed from the data. On average, 2.4 trials were removed per subject.

#### 2.1.5 Data Analysis

The behavioral results from the target detection task were used to determine subject exclusion. Subjects were to be excluded with a detection accuracy under 0.8, but all participants performed above this limit. To evaluate participants' engagement in the task and experiment, mean accuracies and reaction times are reported.

To investigate the course of face‐gender‐specific cortical activity, we used MVPA. Decoding accuracy was determined using the MVPA‐Light and FieldTrip toolboxes (Treder 2020). A linear discriminant analysis (LDA) algorithm was trained and tested on the facial gender of the given data at all time points of the first 1000 ms of the stimulus presentation. Labels for the stimuli were determined based on the CFD (self‐reported gender), but the cortical patterns represent the perceived gender of a face. Cross‐validation was performed with the leave‐one‐out cross‐validation (LOOCV) method, where one trial was used as test data in one iteration. LOOCV was chosen due to the different identities and number of presentations between the experiments, and to keep the two results comparable. Furthermore, several of the previously mentioned studies utilized LOOCV, which allows better comparisons for previous results (Ambrus et al. 2019; Ambrus et al. 2021; Nemrodov et al. 2018). Throughout the results, the average overall accuracy of the algorithm is reported at each time point. Similar to previous related publications, a 60‐ms‐long moving mean window was applied to the decoding accuracy data (Vida et al. 2017). Accuracy values were reported as peak accuracy (±SD) and mean accuracy (±SD) for significant time windows.

Regional decoding was performed to acquire a limited spatial distribution of the activity. EEG electrodes were divided into six regions: left and right frontal, central, and occipital regions (following the methodology of Ambrus et al. 2019; Ambrus et al. 2021).

#### 2.1.6 Statistics

The decoding accuracy of the algorithm was tested against chance (0.5, binary classification) at all time points (every 10 ms) in the first 1000 ms of the stimulus presentation using one‐sample *t*‐tests (two‐tailed, for the possibility of significantly below‐chance algorithm accuracy). Significant time windows were determined using threshold‐free cluster enhancement (TFCE) implemented in the FieldTrip Toolbox, with parameters *height* = 1 and *extent* = 1. Correction due to multiple comparisons was performed against a null distribution created by permutations of 10,000 iterations. The results of the statistical comparison are reported as peak *t* values (*t*_peak_) and the average *t* value over the significant time periods (*t*_mean_). In all cases, unenhanced *t* values are reported. In the case of interrupted significant windows, mean values are calculated using all significant time points. To calculate the effect sizes, we used Cohen's *d*, peak and average (*d*_peak_, *d*_mean_) according to the following formulas (Lakens 2013):

(1) $$d=\frac{t}{\sqrt{N}}$$

(2) $$d=t\times \sqrt{\frac{1}{N1}+\frac{1}{N2}}\;$$

for one‐sample (1) and independent *t*‐test (2), respectively.

### 2.2 Experiment 2

#### 2.2.1 Participants

Experiment 2 included 25 participants (15 females and 10 males [self‐reported], mean age ± SD of 25.70 ± 4.44 years). All participants were healthy, with correct or corrected‐to‐normal vision, and agreed to participate in the experiment voluntarily. The experiment was approved by the Hungarian National Center for Public Health and Pharmacy (NNGYK/49248‐3/2024).

#### 2.2.2 Procedure and Data Acquisition

The same stimulus bank, procedure, and data acquisition methods were used here as in Experiment 1. The difference in Experiment 2 is that we used all 100 stimuli and six repetitions, which reduced the exposure to a single face due to the low repetition number.

In Experiment 2, we used the same MVPA technique, with an LDA algorithm and LOOCV. Decoding accuracies were evaluated, and significant time windows were determined in the same manner as in Experiment 1. Overall decoding accuracy across time was compared between Experiment 1 and Experiment 2 using independent‐samples *t*‐tests (two‐tailed) at each time point with TFCE correction.

Additionally, across‐experiment cross‐validation was performed, where the training data came from the data of all subjects in one experiment, and the test set was set to be the data of one subject from the other experiment in one iteration. Just like previously, statistical significance was determined using independent *t*‐tests and TFCE correction. Direct comparison of the subpopulations was carried out similarly (described in Experiment 1) using independent *t*‐tests and TFCE correction.

### 2.3 Extension of Experiment 2

To keep the experiment balanced between participants’ genders and to raise statistical power, we expanded the number of subjects and recorded an additional 15 participants. This way, 40 volunteers participated in the extended experiment (20 females and 20 males [self‐reported], mean age ± SD of 24.92 ± 4.26 years). Different *k*‐fold cross‐validations were subsequently applied to the data to elaborate on the dynamics of face‐gender decoding using a high number of identities and a high number of participants. The following cross‐validations were applied in all possible permutations:

- *Within‐subject CV*: Within‐subject analysis included 99 identities in the training set, while the gender of 1 identity was tested using all repetitions of the face. This method resulted in 534 training trials and six testing trials for each subject and each iteration.
- *Between‐subject CV*: To understand whether these cortical patterns are consistent between participants, we trained the LDA algorithm on the data of all participants except one, and the algorithm was tested on the excluded subject's data. Between‐subject CV yielded 21,060 training trials and 540 testing trials through 40 iterations.
- *Within‐gender CV*: To investigate an assumed gender difference in face processing based on behavioral data, our next analysis divided the population based on the gender of the participants. During within‐gender analysis, the training set included the data of all participants in the subpopulation except one, which was used to test the algorithm's accuracy, resulting in 10,260 training trials and 540 testing trials through 20 iterations in both subpopulations.
- *Between‐gender CV*: Analogous to the between‐subject analysis, between‐gender cross‐validation was intended to test the general nature of the cortical patterns between participant genders. The training set included the data of a subpopulation, while the algorithm was tested on the data of one participant from the other subpopulation. This way, 10,800 training trials and 540 testing trials were included in all 20 iterations of both subpopulations.

## 3 Results

### 3.1 Experiment 1

#### 3.1.1 Behavioral Results

Accuracy in the detection task of the experiment was high, with a mean ± SD of 0.97 ± 0.03. The range was between 0.9 and 1. The average reaction time was 0.50 ± 0.06 s.

#### 3.1.2 LOOCV Results

As expected, the decoding of facial gender information was successful in Experiment 1. An early time window can be observed between 85 and 475 ms, which is significantly above 0.5 (Figure 3). At this time window, peak accuracy was at 135 ms after the stimulus presentation, with a mean value ± SD of 0.535 ± 0.030. At 135 ms, the unenhanced *t* value was *t*_peak_ (24) = 5.866 (*d*_peak_ = 1.17). In the significant time window, mean accuracy ± SD was 0.515 ± 0.020, while *t*_mean_ (24) = 3.540 (*d*_mean_ = 0.708).

**FIGURE 3:**
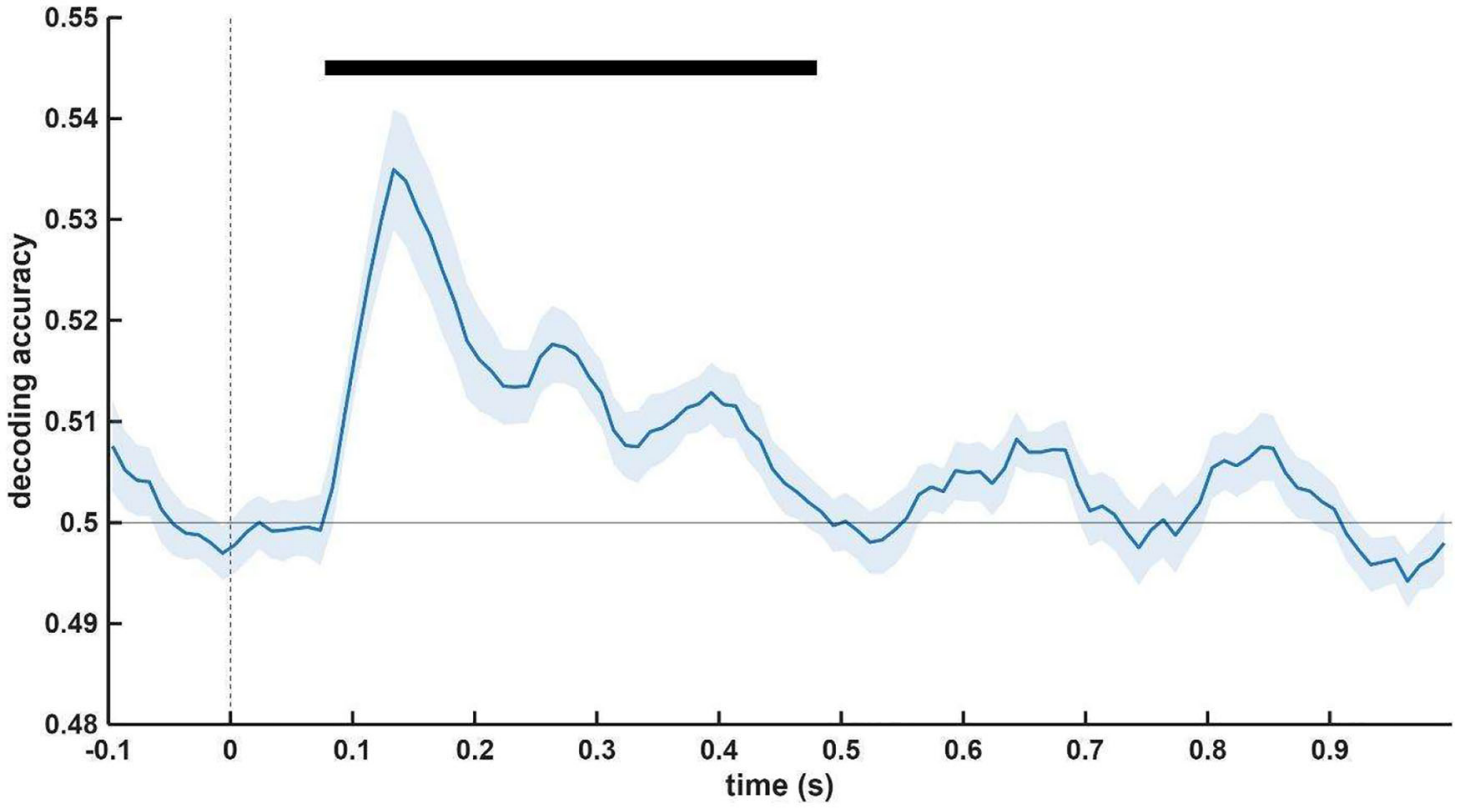
Decoding accuracy across time in Experiment 1. The shaded area marks SEM. Significant periods are indicated by a black bar.

Looking at the decoding accuracy in the previously defined electrode clusters revealed significantly above‐chance time windows predominantly in the right‐side regions. The greatest cluster can be observed in the occipital regions (left: 85–435 ms; right: 85–445 ms). On the left side, peak accuracy (0.535 ± 0.034) was at 135 ms (*t*_peak_ (24) = 3.41, *d*_peak_ = 0.682). In the significant window, mean accuracy was 0.514 ± 0.021 (*t*_mean_ (24) = 3.776, *d*_mean_ = 0.755). On the right, the peak was also at 135 ms (accuracy ± SD = 0.537 ± 0.016), *t*_peak_ (24) = 2.908, *d*_peak_ = 0.582). In the significant window, average accuracy ± SD was 0.513 ± 0.021 (*t*_mean_ (24) = 3.700, *d*_mean_ = 0.740).

Central regions also showed face‐gender‐related patterns, mainly on the right side (95–425 ms). Accuracy reached its peak 125 ms post‐stimulus (peak value ± SD = 0.513 ± 0.016, *t*_peak_ (24) = 2.157, *d*_peak_ = 0.432). The mean accuracy ± SD in the significant time window was 0.505 ± 0.017, with *t*_mean_ (24) = 2.757 (*d*_mean_ = 0.551). Finally, shorter significant time windows also emerged in the right frontal regions between 195 and 885 ms (Figure 4). Peak accuracy falls outside of a significant window 0.415 ms after presentation, with a mean value ± SD of 0.509 ± 0.019. In the significant windows, mean accuracy ± SD was 0.503 ± 0.015, with *t*_mean_ (24) = 2.209 (*d*_mean_ = 0.442).

**FIGURE 4:**
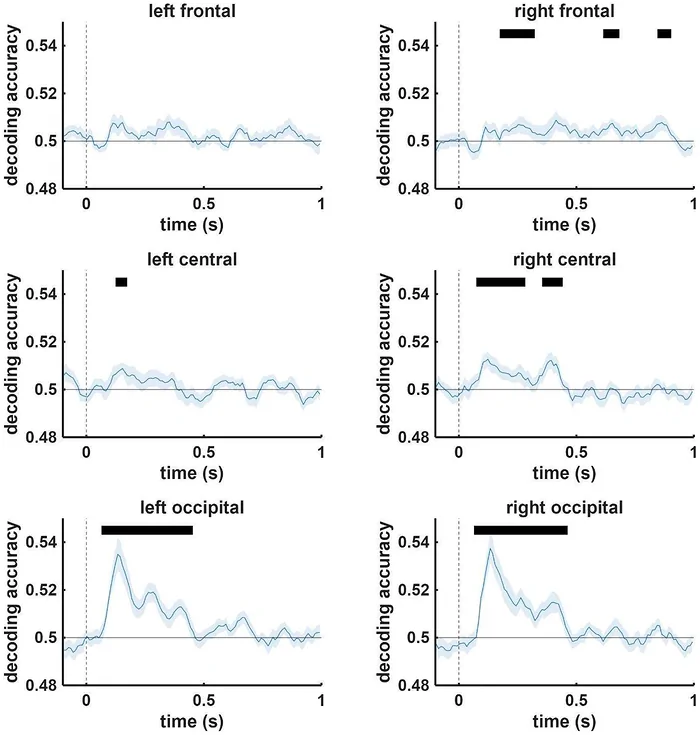
Decoding accuracy across time in Experiment 1 in the six regions described above. The shaded area marks SEM. Significant periods are indicated by a black bar.

### 3.2 Experiment 2

#### 3.2.1 Behavioral Results

Similar to Experiment 1, target detection accuracy was relatively high in Experiment 2 as well (mean ± SD = 0.96 ± 0.03, range: 0.88–1), and reaction time also showed comparable values (mean ± SD = 0.48 ± 0.05).

#### 3.2.2 LOOCV Results

It was also possible to decode face gender from the cortical patterns in Experiment 2, but the significant time window was shorter (95 to 285 ms) compared to the time window found in Experiment 1 (Figure 5). Peak accuracy (accuracy ± SD = 0.517 ± 0.018) was at 145 ms after stimulus presentation (*t*_peak_ (24) = 4.733, *d*_peak_ = 0.947), while in the significant time window, average accuracy was 0.510 ± 0.016 (*t*_mean_ (24) = 3.127, *d*_mean_ = 0.625).

**FIGURE 5:**
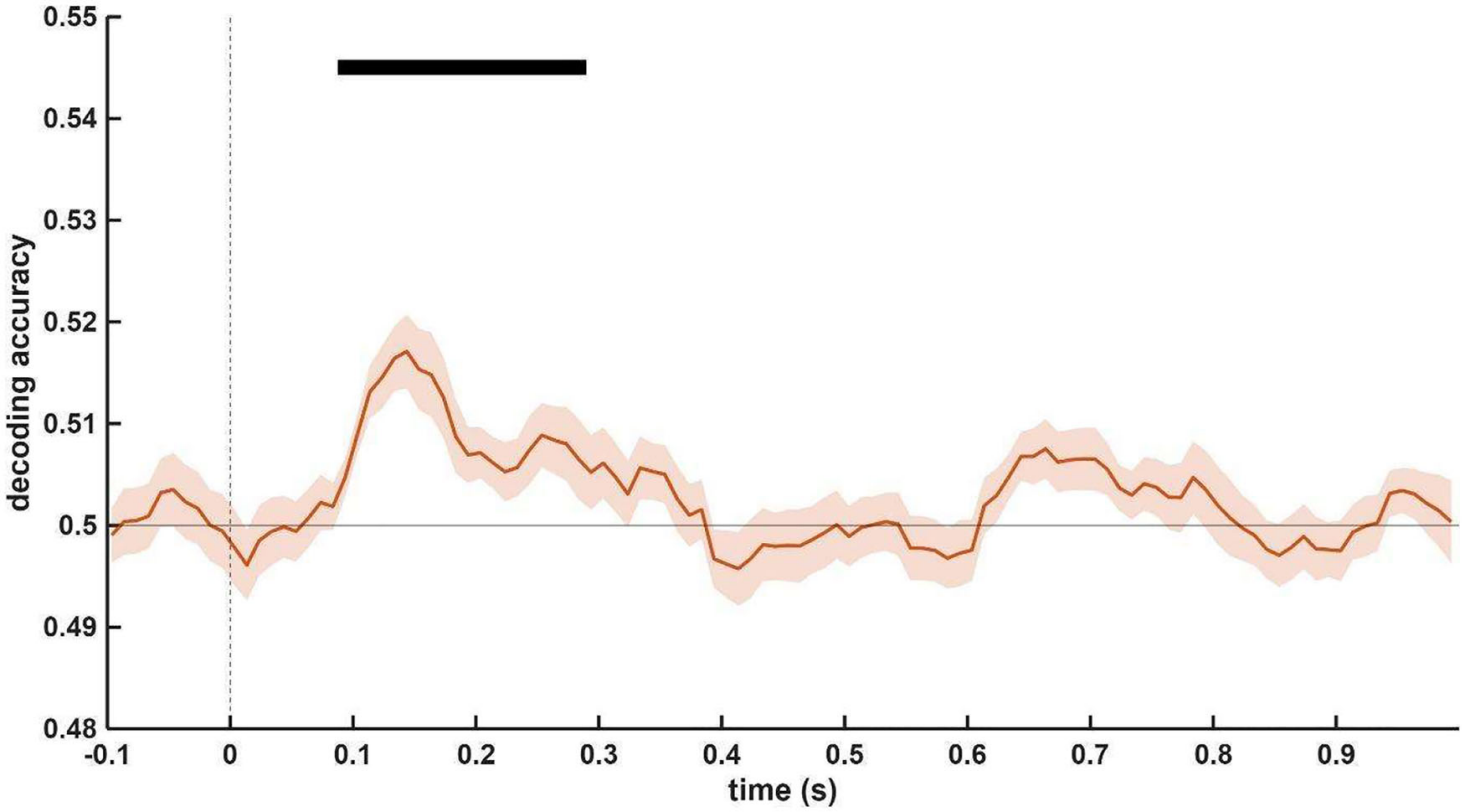
Decoding accuracy across time in Experiment 2. The shaded area marks SEM. Significant periods are indicated by a black bar.

Decoding in the different electrode clusters was also more restricted in Experiment 2. Significant clusters were only observed in the occipital regions with a similar shorter temporal distribution (left: 35–375 ms; right: 85–375 ms; Figure 6). On the left, accuracy reached its peak at 135 ms (accuracy ± SD = 0.520 ± 0.014, *t*_peak_ (24) = 2.037, *d*_peak_ = 0.407). In the significant window, mean accuracy was 0.508 ± 0.015 (*t*_mean_ (24) = 2.86, *d*_mean_ = 0.572). The peak of accuracy on the right was 145 ms after stimulus presentation (accuracy ± SD = 0.523 ± 0.016, *t*_peak_ (24) = 2.446, *d*_peak_ = 0.49). The average accuracy of the significant period was 0.51 ± 0.017 (*t*_mean_ (24) = 3.433, *d*_mean_ = 0.687).

**FIGURE 6:**
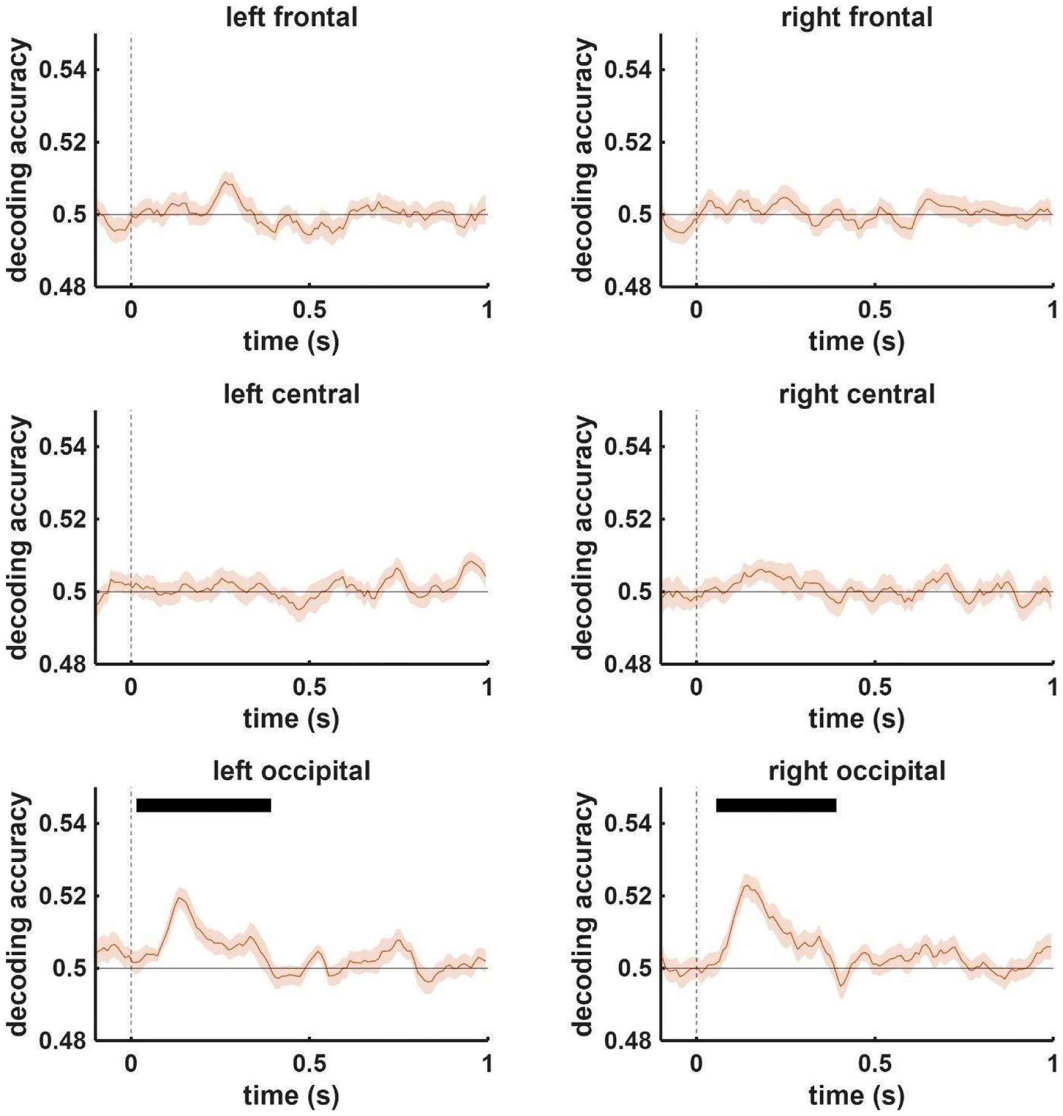
Decoding accuracy across time in Experiment 2 in the six regions described above. The shaded area marks SEM. Significant periods are indicated by a black bar.

### 3.3 Comparison of Experiment 1 and Experiment 2

In the next step of the analysis, LDA accuracy was compared between the two experiments across time. This analysis revealed differences between 95 and 305 ms and 395–405 ms, where the algorithm performed significantly better in Experiment 1 (*t*_mean_ (48) = 2.04, *d*_mean_ = 0.577; Figure 7).

**FIGURE 7:**
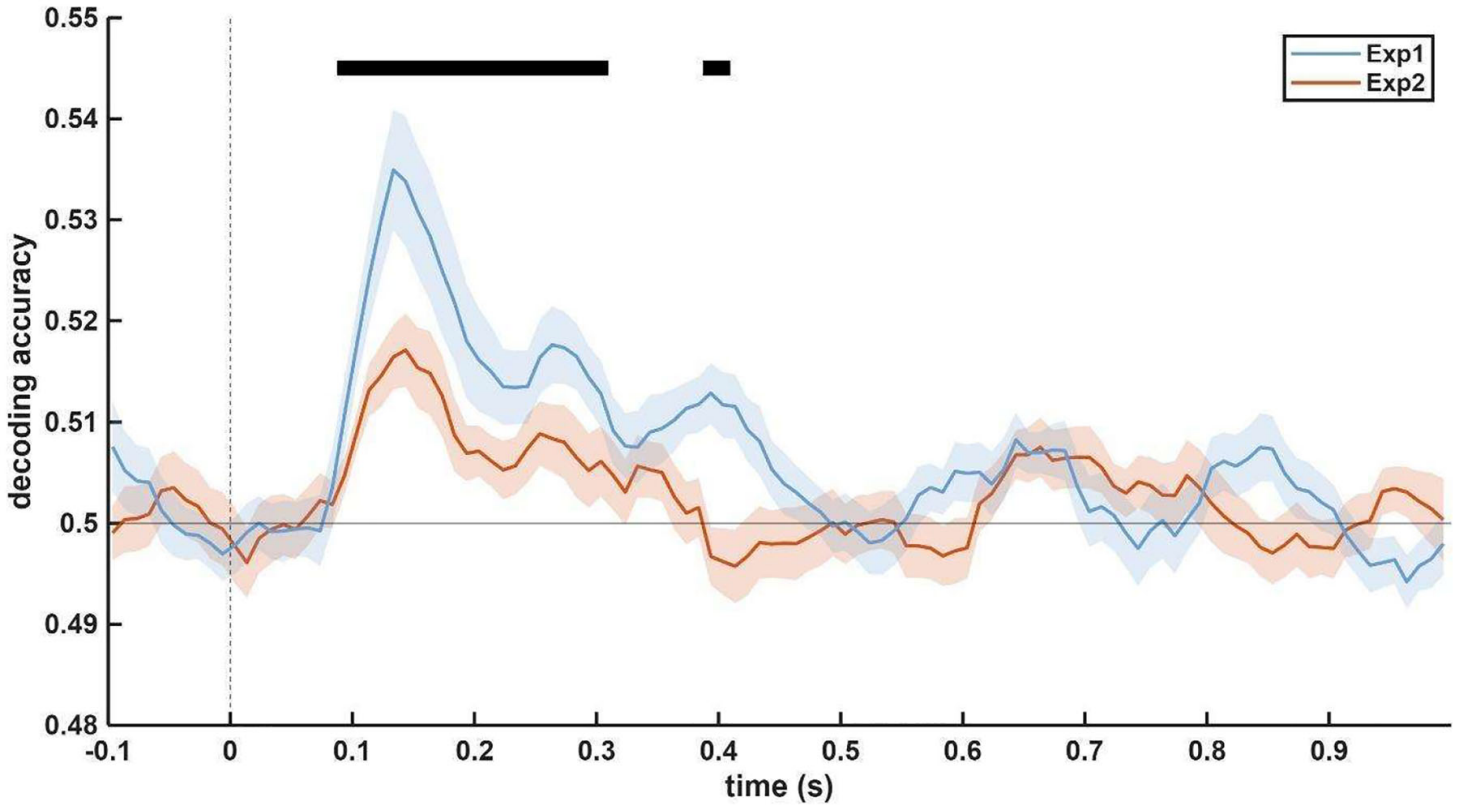
Comparison of the LDA performance in the two experiments across time. The blue line represents Experiment 1, while the orange line represents Experiment 2. The shaded area marks the SEM and the black bar signifies the significant time window.

Since the LOOCV results in an unbalanced representation of one identity (one picture) between the two experiments, the data were reanalyzed with *k*‐fold CV. In this case, in Experiment 1, one female and one male identity were left out of the training set and used as a testing set. Since only six identities were used in Experiment 1, one‐third of the data was used as a testing set. To use the same training‐testing ratio in Experiment 2, one‐third of the trials were used as a testing set as well. This meant that through three iterations, 32–34–34 identities were left out (50% female and 50% male).

The reduction of the training set reduced the LDA performance in both experiments. In Experiment 1, a small, significant window appeared between 375 and 425 ms, while no window emerged in Experiment 2. The comparison of the two experiments resulted in no significant time window either (Figure 8). In the data of Experiment 1, peak accuracy falls outside of the significant time window, at 135 ms (accuracy ± SD = 0.517 ± 0.289). In the significant time window, mean accuracy was 0.508 ± 0.0126 (*t*_mean_ (24) = 2.881, *d*_mean_ = 0.576). In Experiment 2, peak decoding accuracy occurred at 155 ms post‐stimulus with a value of 0.511 ± 0.251.

**FIGURE 8:**
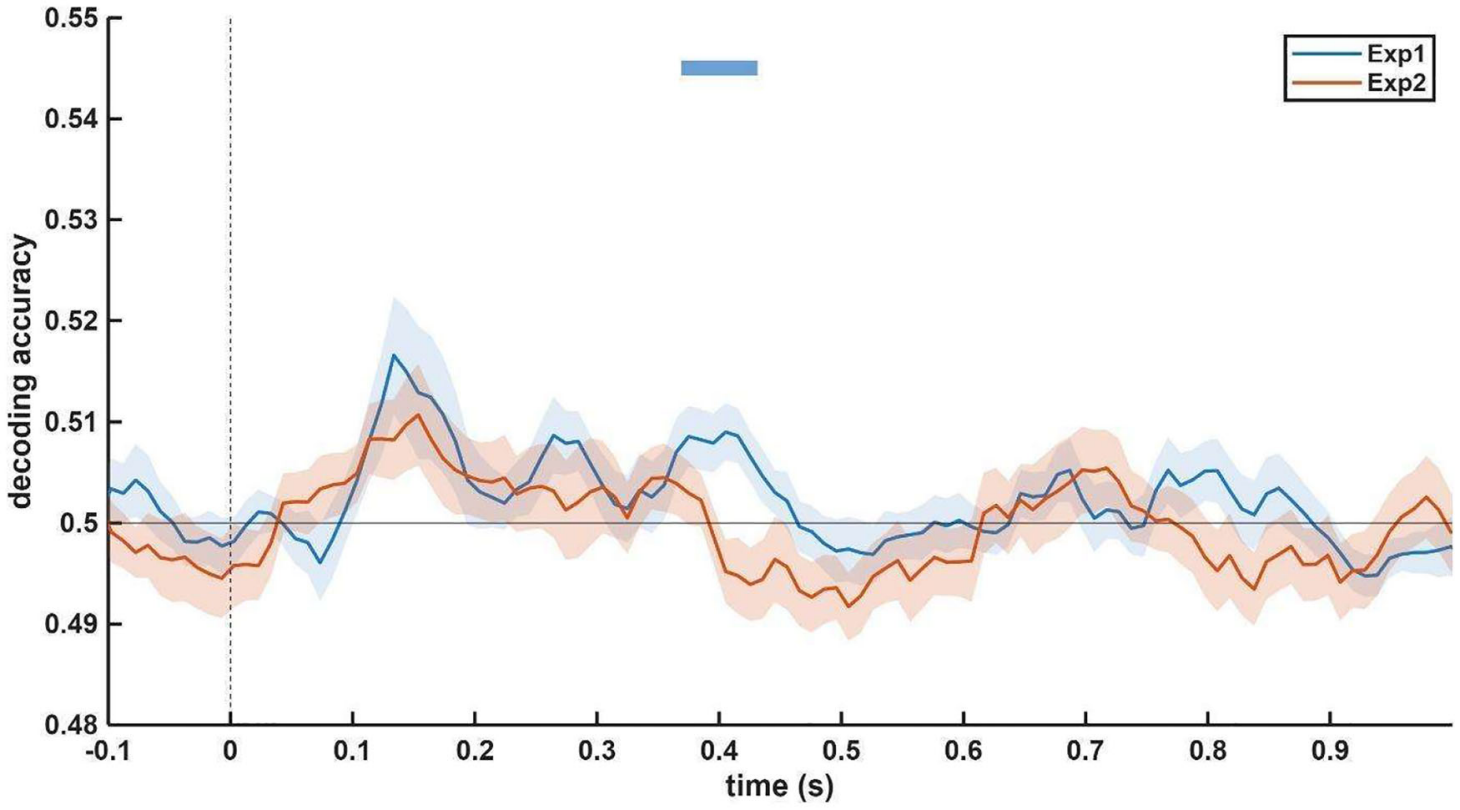
Comparison of the LDA performance in the two experiments across time using *k*‐fold cross‐validation. The blue line represents Experiment 1, while the orange line represents Experiment 2. The shaded area marks SEM, and the bar with the corresponding color represents the significant time window.

To further investigate the common neural patterns in gender decoding, we performed an across‐experiment cross‐validation. Training on Experiment 2 and testing on Experiment 1 revealed a significant window between 105 and 265 ms. In the window, mean accuracy was 0.509 ± 0.010 with *t*_mean_ (24) = 2.816 (*d*_mean_ = 0.563). Peak accuracy was reached at 135 ms, with a value of 0.513 ± 0.010, where *t*_peak_ (24) = 3.255 (*d*_peak_ = 0.651). Similarly, training on Experiment 1 and testing on Experiment 2 resulted in an above‐chance period between 105 and 255 ms (Figure 9), with a peak at 135 ms (peak accuracy ±SD = 0.518 ±0.012, *t*_peak_ (24) = 7.538, *d*_peak_ = 1.508). In the significant window, mean accuracy was 0.511 ± 0.010, with *t*_mean_ (24) = 4.789 and *d*_mean_ = 0.958.

**FIGURE 9:**
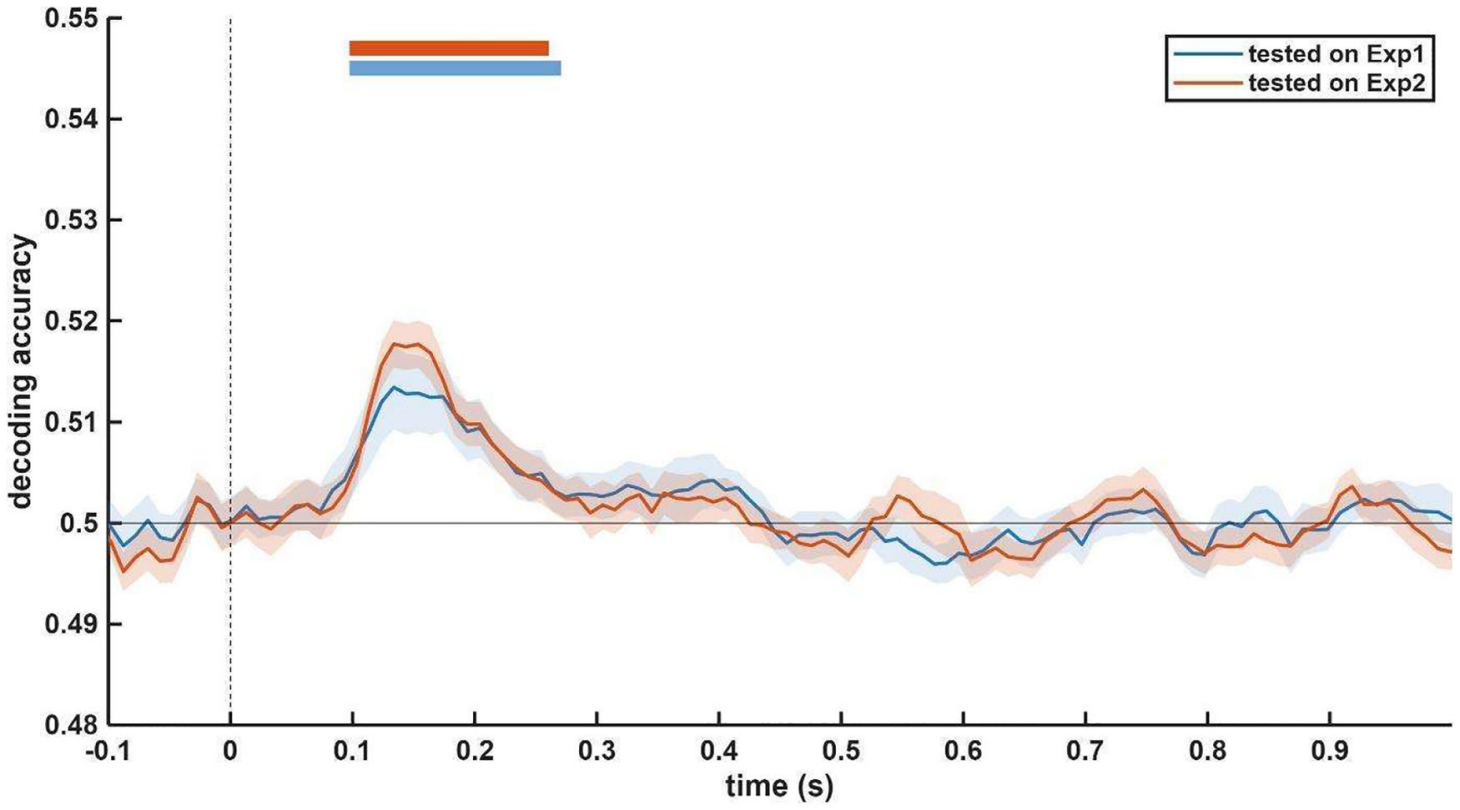
Decoding accuracy across time using across‐experiment cross‐validation. Significant periods are marked by the bars with corresponding colors. The data from Experiment 2 was used as a training set, while testing was performed on the data of one subject from Experiment 1 (blue line). The training set was the data from Experiment 1, while the testing set was Experiment 2 (orange line).

### 3.4 Extension of Experiment 2

Behavioral results remained high with the extension. Mean accuracy was above 0.8 for all subjects (mean ± SD = 0.97 ± 0.03, range: 0.88–1). Reaction time was fairly similar to the above‐reported results (mean ± SD = 0.49 ± 0.05 s).

A within‐subject CV, where one out of 100 identities was used for the *k*‐fold cross‐validation, revealed an early time window. Above‐chance decoding was detectable between 75 and 355 ms post‐stimulus, unsurprisingly, almost identical to LOOCV analysis. At its peak (155 ms), accuracy was 0.514 ± 0.014 (*t*_peak_ (39) = 5.172, *d*_peak_ = 0.818). On average in the window, accuracy ± SD was 0.507 ± 0.014 (*t*_mean_ (39) = 3.044, *d*_mean_ = 0.481). Between‐subject CV revealed a more restricted significant time window from 85 to 315 ms (mean accuracy ± SD = 0.508 ± 0.011, *t*_mean_ (39) = 4.088, d_mean_ = 0.646), characterized by an identical onset but a shorter duration compared to within‐subject CV, along with an additional brief (20 ms) significant period around 740 ms (Figure 10). Peak accuracy can be observed at 165 ms after stimulus presentation (accuracy ± SD = 0.516 ± 0.011), *t*_peak_ (39) = 8.139, *d*_peak_ = 1.287).

**FIGURE 10:**
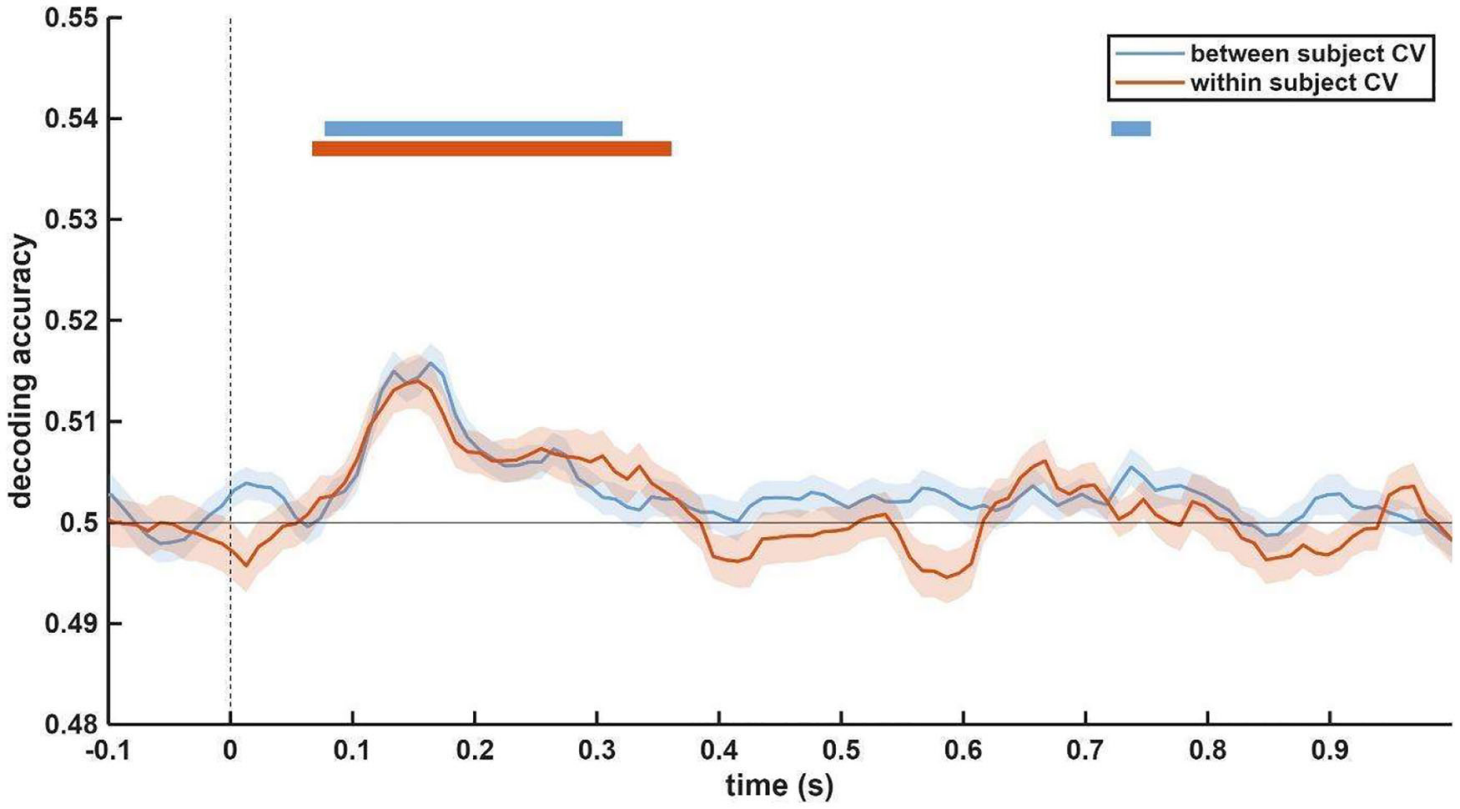
Decoding accuracy across time with within‐subject CV and between‐subject CV. The training set included all identities except one presented to one subject, which was subsequently used as a test set during within‐subject CV (orange line). For the between‐subject CV, the training set was determined as data from all subjects except one, which was used as a test set (blue line). Significant time windows are indicated by the corresponding bars.

Using within‐gender CV revealed a temporal distribution for the above‐chance period between 95–295 ms in the case of the male subpopulation (mean accuracy ± SD = 0.509 ± 0.01, *t*_mean_ (19) = 3.562, *d*_mean_ = 0.796) and 115–275 ms in the case of the female subpopulation (mean accuracy ± SD = 0.508 ± 0.010, *t*_mean_ (19) = 3.429, *d*_mean_ = 0.767). In the male subpopulation, peak accuracy was at 135 ms (peak accuracy ± SD = 0.517 ± 0.011, *t*_peak_ (19) = 5.06, *d*_peak_ = 1.131), while in the female subpopulation the peak can be observed at 165 ms after stimulus presentation (peak accuracy ± SD = 0.510 ± 0.011, *t*_peak_ (19) = 4.596, *d*_peak_ = 1.028). This distribution is very similar to the between‐subject CV, which can be attributed to the similarities between the two cross‐validations, as within‐gender CV is fundamentally a between‐subject CV within a subpopulation. Direct comparison of the two within‐gender subpopulations revealed no significant difference in decoding accuracy (Figure 11, top).

**FIGURE 11:**
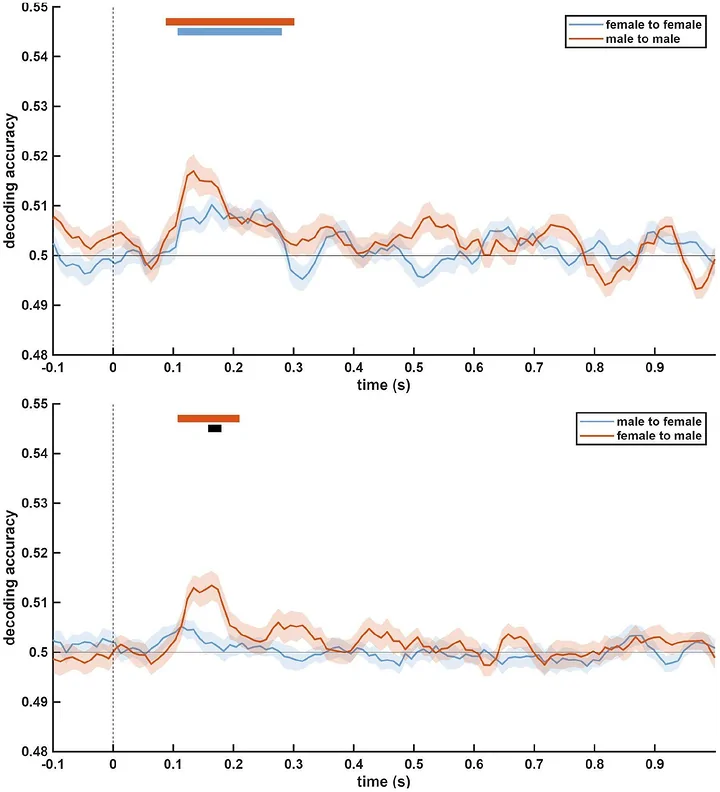
Top: Decoding accuracy across time using within‐gender CV. The cross‐validation used either the male or female subpopulation, with all subjects except one used as the training set and the remaining subject tested. The blue line represents the analysis where only the female subpopulation was used, while the orange line represents the male subpopulation. Bottom: Decoding accuracy with between‐gender CV. The training set was determined as one subpopulation, either male or female. The test set was the data of one subject from the other subpopulation. The blue line represents the analysis where male subjects were used as a training set, and the model was tested on female participants. The orange line represents the opposite; the model was trained on the data of female participants and was tested on the data of male participants. The black line represents the significant time window after the comparison of the accuracies of the two cross‐validations.

Finally, the shortest significant time window was observed with between‐gender CV, ranging from 115 to 205 ms (mean accuracy ± SD = 0.51 ± 0.01, *t*_mean_ (19) = 3.537, *d*_mean_ = 0.791) when the model was trained on female data and tested on male data. Peak decoding was at 165 ms (peak accuracy ± SD = 0.513 ± 0.009, *t*_peak_ (19) = 4.542, *d*_peak_ = 1.017). When male data were used for training and tested on female data, no significant clusters emerged (peak decoding at 115 ms, peak accuracy ± SD = 0.505 ± 0.01), Figure 11, bottom). Direct comparison revealed that training on female data and testing on male data resulted in significantly higher accuracy in a small time window (165–175 ms after stimulus presentation) than training on male data and testing on female data (*t*_mean_ 38 = 3.244, *d*_mean_ = 1.026).

## 4 Discussion

In this study, we investigated the effect of face recognizability and exposure on cortical decoding using different experimental setups. We hypothesized that previously reported MVPA/RSA results on facial sex and gender decoding are influenced by the utilized paradigm. To test this hypothesis, we carried out two experiments where exposure to a face was altered using the number of presented identities and the number of presentations per identity.

In Experiment 1, we employed a paradigm commonly favored in previous research, characterized by a small number of identities coupled with high repetition numbers. Using these methods, we observed a time window where facial gender information can be decoded from the cortical pattern. The temporal distribution of this window was similar to the literature, with a very short latency of 80 ms and the latest significant periods as late as 880 ms. The regional analysis of the different cluster electrodes also showed a spatial distribution similar to previous reports, where identity information of familiar faces emerged over the occipital, central, and frontal regions, predominantly on the right side (Ambrus et al. 2019).

During Experiment 2, the ratio of identities and presentation numbers was switched, reducing exposure to one identity; other aspects of the stimulus presentation remained unchanged. This modification resulted in a more confined window where the perceived gender of a facial stimulus can be decoded above chance. The distribution of the above‐chance decoding was restrained not only temporally but spatially as well, since above‐chance decoding was only detectable in the occipital regions. The comparison of the two experiments revealed that the identity/repetition ratio affects the decoding accuracy and thus the cortical patterns not only temporally and spatially but most likely in the signal‐to‐noise ratio as well, since the LDA algorithm performed significantly better in the early time windows in Experiment 1 compared to Experiment 2. Furthermore, with across‐experiment cross‐validation, we found a time window between 100 and 260 ms that appears to contain shared neural activity patterns between the two experiments.

After establishing the effect of the different paradigms, we extended the number of subjects in Experiment 2. With this extension, we were able to investigate the neural pattern and gender decoding of non‐recognizable faces. We took the opportunity to use the high number of participants to find shared cortical patterns across subjects and the genders of observers. We identified significant time windows using different k‐fold cross‐validations, which suggests the existence of general or abstract patterns in the activation of the cortex that are detectable by MVPA techniques.

### 4.1 The Decoding of Facial Gender Information

Numerous publications have investigated the temporal distribution of cortical patterns related to facial sex or gender processing. Previous studies have identified the time window when the perceived gender of a presented face stimulus can be determined accurately. Most studies concluded that decoding accuracy emerges around 100 ms after stimulus presentation, with the earliest evidence found around 50 ms (Nemrodov et al. 2018). It is also shared across the studies that the decoding windows usually last about 600–700 ms, ending around 700–800 ms after the presentation of a stimulus. The regional analyses also revealed the occipital regions and the greater engagement of right central and frontal clusters (Ambrus et al. 2019). Besides the similarities in the results, many aspects of the applied paradigms are shared as well. Many studies use a low number of identities coupled with a lot of repetitions, for example, 16 identities and 140 repetitions per identity (Dobs et al. 2019), four identities and 400 repetitions per identity (Ambrus et al. 2019), four identities and 1300 repetitions per identity (Nemrodov et al. 2016). The use of high repetition numbers was inevitable in these studies since most investigated several facial features, like gender, identity, age, familiarity, and so on. Additionally, one identity included several stimuli/pictures, which boosted the variance in these paradigms. We already have evidence that changes in the neural pattern and decoding can emerge due to the perceptual familiarization process (Ambrus et al. 2021). Although perceptual familiarization has involved the smallest changes compared to media and personal familiarization processes, this effect cannot be neglected. Familiarity can be decoded from cortical activity, and the effect of familiarity on the face‐processing mechanism has been established (Kovács 2020).

So far, to our knowledge, only one other study has investigated identity‐related cortical patterns using a high number of identities with a low repetition number (Vida et al. 2017). They reported a narrower significant window (100–400 ms), although the smaller sample size in their study might have contributed to this restricted timeframe. Our results align well with the literature, where perceptually familiarized faces induced gender‐related cortical patterns to remain detectable for longer, while in the case of completely unfamiliar faces, these patterns are short‐lived. Although many aforementioned studies investigated the decoding of familiarity, our findings suggest that in the utilized paradigms even unfamiliar faces can evoke more stable cortical representations through highly repeated exposures. Based on our results, it is possible that the presented faces can acquire some degree of familiarity proportional to the number of repeats, although the level of acquired familiarity was not measured directly in the current study. These results also indicate that repeated exposure affects seemingly unrelated features, such as perceived gender.

We already have evidence that expectation can improve stimulus representation (Kok et al. 2012) and that familiar items can elicit superior perceptual performance (Manahova et al. 2018). The comparison of the two experiments also indicates that faces with a greater degree of familiarity elicit greater decoding accuracies. This result could suggest a better signal‐to‐noise ratio in the face‐processing network; however, further investigation is required for definite proof due to the differences in Experiment 1 and Experiment 2. Namely, this difference is only detectable using LOOCV, which could result in overrepresentation of one identity during training in Experiment 1.

To investigate this, we reanalyzed the data with *k*‐fold CV, where one‐third of the trials were used for testing. This method drastically reduced the accuracy of the decoding algorithms in both experiments and removed the significant difference between the two. Nevertheless, significant decoding above chance was still achieved in Experiment 1, even with this reduced training set, whereas Experiment 2 did not show significant decoding accuracies. The reduction in decoding accuracy in the *k*‐fold CV can be attributed to the reduced training set size (Fukunaga and Hayes 1989; Jain et al. 2000), but the lack of identity information could have also contributed to the decreased decoding performance. This finding raises the question of how identity information found in the training set affects the decoding performance, considering prior studies also employed LOOCV (Ambrus et al. 2019; Ambrus et al. 2021; Nemrodov et al. 2018) or randomized *k*‐fold CV (Dobs et al. 2019), which could potentially inflate accuracy results of gender decoding. Whether this potential contamination meaningfully skews performance metrics requires further investigation.

### 4.2 Shared Neural Patterns in Facial Gender Processing

There is a growing number of studies investigating the neural patterns associated with different aspects of perception and cognitive functions using decoding techniques. These studies usually use train and test sets within a subject, which is highly specific to personal cortical activity but does not provide any information about common patterns in the population. Recent studies have started to examine these shared activities by using train and test sets across subjects or even experiments (Kaplan et al. 2015), and several cognitive functions have been identified that possess common cortical patterns, such as memory and recall (Ambrus 2024), the reward system (Wake and Izuma 2017), or familiarity (Dalski et al. 2022; Li et al. 2022).

Shared neural patterns in face perception have been identified as well. It has been established that face familiarity elicits shared activity 200 ms after stimulus presentation (Ambrus 2024; Dalski et al. 2022). The investigation of the processing of different facial expressions and emotions has also led to the observation of shared neural patterns.

A previous study has investigated the common patterns in cortical activity regarding facial gender information and its perception (Li et al. 2022). Their results showed that shared patterns could be detected in the EEG data around 150–400 ms after stimulus presentation. This finding is comparable to our between‐subject CV results, which resulted in a 200‐ms time window starting 100 ms after stimulus presentation. The difference in the length of these shared patterns could be attributed to differences in the paradigm, as in their case, eight presented identities were used compared to the 100 used in this current study.

Our results confirmed and expanded upon previous findings by utilizing distinct, non‐overlapping subpopulations for training and testing. Behavioral and neuroimaging evidence already indicates that face perception differs by gender (Ino et al. 2010). Here, although shared cortical patterns were identified across subpopulations, between‐gender cross‐validation yielded a noticeably shorter significant decoding window (≈80 ms) than within‐gender cross‐validation. This discrepancy strongly suggests the presence of gender‐specific neural patterns. Furthermore, comparing cross‐gender decodings revealed a brief window centered at 165–175 ms post‐stimulus. This tightly aligns with the N170 ERP component, an established marker of early face processing known to exhibit gender‐dependent cortical modulation (Sun et al. 2010) and distinct lateralization dynamics, where male subjects display right‐sided asymmetry while female subjects demonstrate bilateral or left‐sided engagement (Proverbio 2021).

### 4.3 Limitations of the Current Study

The paradigms and findings presented here utilized different levels of exposure to a singular identity, resulting in altered cortical processing patterns. This change in processing could have potentially emerged due to a change in the degree of familiarity; however, familiarity was not directly measured. This inference is nonetheless supported by prior research demonstrating “perceptual familiarization” following repeated passive exposure to face stimuli (Ambrus et al. 2021), consistent with the cortical changes observed in our data. Future studies would benefit from direct measurements of familiarity to validate these observations.

A further limitation arises from parameter discrepancies between Experiment 1 and Experiment 2. Due to differences in identity sets and exposure counts, no single cross‐validation method allows for a perfect comparison. A leave‐one‐trial‐out method balances training and trial counts across experiments, but leads to the overrepresentation of individual identities. Conversely, a leave‐one‐identity‐out method creates unbalanced training‐to‐testing ratios between experiments. To balance this, we equalized the number of training and testing trials across both experiments. This design required using only two‐thirds of our dataset during training, thus potentially increasing classification error (Fukunaga and Hayes 1989; Jain et al. 2000).

Finally, trials were categorized according to Chicago Face Database (CFD) self‐reported gender labels provided by the stimulus models, while our decoding reflects the perceived gender. Nevertheless, potential discrepancies between self‐reported and perceived gender in the CFD have previously been evaluated, demonstrating high overlap (Ma et al. 2015). While gender and sex representations might be quite distinct in the neural space, it was not possible to control for these differences in the current study due to the nature of the image dataset. Future work might focus on this issue by utilizing a more diverse set of images, treating the two constructs as separate independent variables.

## 5 Conclusion

The current study investigated recognizability and its effect on maintaining facial gender information longer in the cortex. Our findings confirmed previous suspicions that using face perception paradigms with a small number of identities can greatly affect the processing of other facial aspects, in our case, the processing of perceived gender information. By employing a large number of identities, we effectively reduced exposure, which correlated with shorter significant decoding time windows and lower decoding accuracies, suggesting a diminished signal‐to‐noise ratio in the underlying cortical activity. This observation, however, came from data where identity was overrepresented. By reducing the training data, identity information was balanced out, at the same time greatly reducing the decoding accuracies in both experiments. Despite this discrepancy between the different cross‐validation methods, our results highlight the impact of study parameters, more closely the exposure and its effects.

Furthermore, we extended the participants in the second part of the study to investigate the shared neural patterns in the population and between subpopulations (male and female). Both analyses identified significant time windows, suggesting the existence of common cortical patterns regarding facial gender processing, even between distinct subpopulations.

## Author Contributions

**Szabolcs Sáringer**: data curation, formal analysis, investigation, methodology, software, visualization, writing – original draft. **András Benyhe**: data curation, investigation, software, validation, writing – review and editing. **Péter Kaposvári**: conceptualization, funding acquisition, methodology, project administration, supervision, validation, writing – review and editing.

## Funding

This publication was funded by the University of Szeged Open Access Fund (7924).

## Conflicts of Interest

The authors declare no conflicts of interest.

## Ethics Statement

All participants gave written informed consent before registration, and the study protocol was approved by the Hungarian National Center for Public Health and Pharmacy (NNGYK/49248‐3/2024).

## Data Availability

The data and scripts used for analyses in the current study are available in the Open Science Framework repository (https://osf.io/p593q/).
